# Smad3 initiates oxidative stress and proteolysis that underlies diaphragm dysfunction during mechanical ventilation

**DOI:** 10.1038/s41598-017-11978-4

**Published:** 2017-11-06

**Authors:** Huibin Tang, Catherine L. Kennedy, Myung Lee, Yang Gao, Hui Xia, Francesca Olguin, Danielle A. Fraga, Kelsey Ayers, Sehoon Choi, Michael Kim, Amir Tehrani, Yasser A. Sowb, Thomas A. Rando, Joseph B. Shrager

**Affiliations:** 1 Division of Thoracic Surgery, Department of Cardiothoracic Surgery, Stanford University School of Medicine, Palo Alto, CA USA; 2 VA Palo Alto Healthcare System, Palo Alto, CA USA; 3 Respiratory Management Technologies, LLC., San Francisco, CA USA; 4 Paul F. Glenn Laboratories for the Biology of Aging and Department of Neurology and Neurological Sciences, Stanford University School of Medicine, Stanford, CA USA; 5 Present Address: University of Maryland School of Medicine, Baltimore, MD USA; 6 Present Address: Department of Thoracic-cardio Surgery, First Affiliated Hospital of PLA General Hospital, Beijing, China; 7 Present Address: Department of Thoracic and Cardiovascular Surgery, Asan Medical Center, Seoul, Korea

## Abstract

Prolonged use of mechanical ventilation (MV) leads to atrophy and dysfunction of the major inspiratory muscle, the diaphragm, contributing to ventilator dependence. Numerous studies have shown that proteolysis and oxidative stress are among the major effectors of ventilator-induced diaphragm muscle dysfunction (VIDD), but the upstream initiator(s) of this process remain to be elucidated. We report here that periodic diaphragm contraction via phrenic nerve stimulation (PNS) substantially reduces MV-induced proteolytic activity and oxidative stress in the diaphragm. We show that MV rapidly induces phosphorylation of Smad3, and PNS nearly completely prevents this effect. In cultured cells, overexpressed Smad3 is sufficient to induce oxidative stress and protein degradation, whereas inhibition of Smad3 activity suppresses these events. In rats subjected to MV, inhibition of Smad3 activity by SIS3 suppresses oxidative stress and protein degradation in the diaphragm and prevents the reduction in contractility that is induced by MV. Smad3’s effect appears to link to STAT3 activity, which we previously identified as a regulator of VIDD. Inhibition of Smad3 suppresses STAT3 signaling both *in vitro* and *in vivo*. Thus, MV-induced diaphragm inactivity initiates catabolic changes via rapid activation of Smad3 signaling. An early intervention with PNS and/or pharmaceutical inhibition of Smad3 may prevent clinical VIDD.

## Introduction

Mechanical ventilation (MV) is widely used to support patients’ oxygenation and ventilation during surgical procedures and in intensive care units. Weaning from MV is frequently difficult, and failure to wean may result in prolonged ventilator dependence, which dramatically increases subsequent complications including ventilator-associated pneumonia, thereby increasing overall morbidity and mortality^1,2^. A substantial clinical literature establishes that a major contributor to prolonged ventilator dependence is the progressive weakness of the major inspiratory muscle, the diaphragm, which develops rapidly after the institution of MV^3–12^. Mechanistic studies in human and animal models have shown that MV with full ventilator support leads to time-dependent proteolytic and functional changes in diaphragm muscle^13–17^ which are tightly linked to the occurrence of oxidative stress^18–25^. The resulting progressive reduction in diaphragm contractility and muscle mass has been coined ventilation-induced diaphragm dysfunction (VIDD).

Activation of the proteasome system, documented by evidence of increased protein oxidation and protein ubiquitination, is one of the key contributors to proteolysis in MV diaphragm^26–28^. It has been shown that the upstream regulatory signaling of these proteolytic events is linked to mitochondrial oxidative stress (MOS)^21,23^ and JAK-STAT signaling^29,30^. MOS is elevated in MV diaphragm in both animal models and human samples^23,27^, and *in vitro* experiments have demonstrated that MOS is sufficient to trigger proteolytic processes^23^. Antioxidants have been shown to prevent diaphragm muscle protein degradation associated with MV and in some studies to facilitate weaning patients from the ventilator^31–34^. On the other hand, JAK-STAT signaling seems to serve as an amplifier of MOS: oxidative stress is able to activate STAT3, and activated STAT3 is able to induce protein degradation and oxidative stress. Inhibition of JAK-STAT signaling significantly reduces oxidative stress, muscle atrophy and VIDD^29,30^.

In addition to the above molecular and chemical signaling that is altered in MV diaphragm muscle, a physical signal – muscle electrical activity–is also altered. Mechanical ventilation in a full support mode essentially abrogates electrical activity, eliminating contraction of the diaphragm muscle^35–38^. Since muscle activity is known to regulate protein turnover and muscle mass, and since muscle inactivity accelerates protein degradation, it is reasonable to speculate that diaphragm inactivity during MV initiates the process that leads to VIDD. Indeed, reducing MV-induced diaphragm inactivity by use of assisted modes of MV^39^, or inducing diaphragm muscle activity during MV, show promise in preventing VIDD. Electrical stimulation of either diaphragm muscle or phrenic nerve during MV has been shown to recover type II muscle fiber cross sectional area in sheep^40^, improve diaphragm muscle contractility in rats^41^, reduce lactate release in piglet^42^, and increase mitochondrial respiration rates and contractility in humans^10,43^. Transvenous phrenic nerve stimulation has also been shown to mitigate ventilation-induced diaphragm atrophy^44^.

However, it remains largely unclear how this electrical signal is linked to the chemical events that occur in MV diaphragm muscle, resulting in VIDD. We therefore focus, in the current study, on the molecular link between the physical signal (muscle activity) and the subsequent effectors of VIDD. We study this link by examining the changes in MV diaphragm that occur early in the evolution of this syndrome – the time-point at which the atrophy phenotype has not yet developed but at which the effectors have been set into motion – with the intention of identifying the early responsive upstream initiator(s).

Our model for this work is phrenic nerve stimulation (PNS) in short-term, mechanically ventilated rats. In our rats receiving PNS, the phrenic nerve is accessed in the neck and stimulated once every 5 breaths, with the resultant single diaphragm contraction replacing one ventilator breath. This periodic stimulation pattern was sufficient to impressively prevent MV-induced ubiquitin proteasome system (UPS) activity and oxidative stress. Most interestingly, we discover that the transcription factor Smad3 is significantly phosphorylated in MV diaphragm, and that this phosphorylation is nearly completely inhibited by PNS. The phosphorylation/activation of Smad3 is seen even at only 6 hours after MV and leads to nuclear enrichment. We also show that elevated Smad3 activity in muscle cells is sufficient to induce protein ubiquitination, protein oxidation, and MOS; and that inhibition of Smad3 phosphorylation in muscle with SIS3, both *in vitro* and *in vivo*, leads to suppression of protein oxidation and UPS activity as well as prevention of MV-associated reduction in the specific force of contraction of diaphragm muscle. Thus, we demonstrate that Smad3 is an activity-dependent sensor whose activation initiates inactivity-induced diaphragm muscle protein degradation and diaphragm dysfunction during MV.

## Results

### Periodic, synchronized phrenic nerve stimulation (PNS) prevents MV-induced oxidative stress and ubiquitin proteasome activity

**Figure 1:**
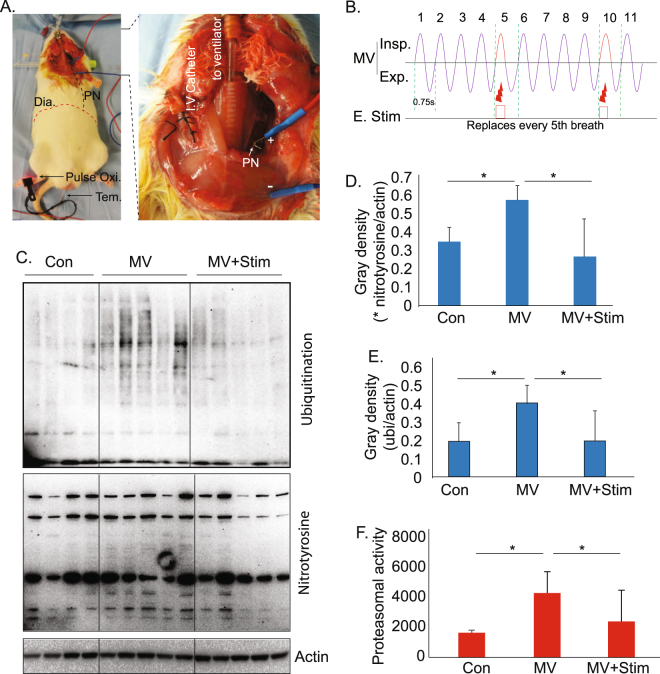
Phrenic nerve stimulation suppresses oxidative stress, protein ubiquitination, and proteasomal activity, which are each elevated in mechanically ventilated diaphragm. (**A**) Rat MV and PNS set-up allowing full monitoring and control of physiology to maintain homeostasis. Dia – diaphragm, PN – phrenic nerve, Tem – rectal temperature probe, IV – intravenous. (**B**) A diaphragm contraction is delivered via PNS to replace every fifth ventilator-delivered breath. Insp – inspiration, Exp – expiration, E Stim – electrical stimulation of the phrenic nerve. (**C**) Western blot analysis of diaphragm muscles in control, after 12 h MV, and after 12 h MV+Stim. Con – control; Stim – phrenic nerve stimulation. Note, the hemi-diaphragm muscles ipsilateral to the stimulation were harvested for analysis. (**D**,**E**) Quantitation of protein levels of ubiquitinated and oxidized proteins in control (n = 4), 12 h MV (n = 5), and 12 h MV + Stim (n = 5) diaphragm. (**F**) Proteasomal activity (chymotrypsin-like) in control (n = 4), 12 h MV (n = 5), and 12 h MV + Stim (n = 5) diaphragm. Data information: In (**D**–**F**), data are presented as mean ± standard deviation. *p < 0.05 (One way ANOVA analysis followed by a post-hoc Tukey HSD Test).

To determine if MV-dependent diaphragm inactivity is directly linked to the elevated ubiquitin-proteasome activity and oxidative stress seen in MV diaphragm muscle, we examined whether diaphragm muscle activity evoked by PNS can prevent this MV-associated activation of ubiquitin-proteasome activity and oxidative stress. To prevent a situation in which a diaphragm contraction induced by PNS might induce either muscle fatigue or eccentric contraction causing muscle injury, we stimulated the diaphragm in a periodic (once every 5 ventilator breaths) and synchronized (with the stimulated contraction timed to occur during a single, skipped ventilator breath) fashion (Fig. 1A and B). This stimulation parameter was carried out unilaterally, continuously during 12 hours of MV.

Consistent with previous reports^18,26,28^, MV significantly increases protein ubiquitination and oxidation (Fig. 1C–E), as well as ubiquitin proteasome activity (Fig. 1F) in diaphragm muscle. Our periodic, synchronized PNS protocol inhibited MV-induced protein ubiquitination, oxidation and proteasome activity to levels not different from non-ventilated controls (Fig. 1C–F). These data indicate that muscle activity plays a major role in MV-induced pathogenic changes.

### Smad3 is rapidly activated in mechanically ventilated diaphragm muscle

**Figure 2:**
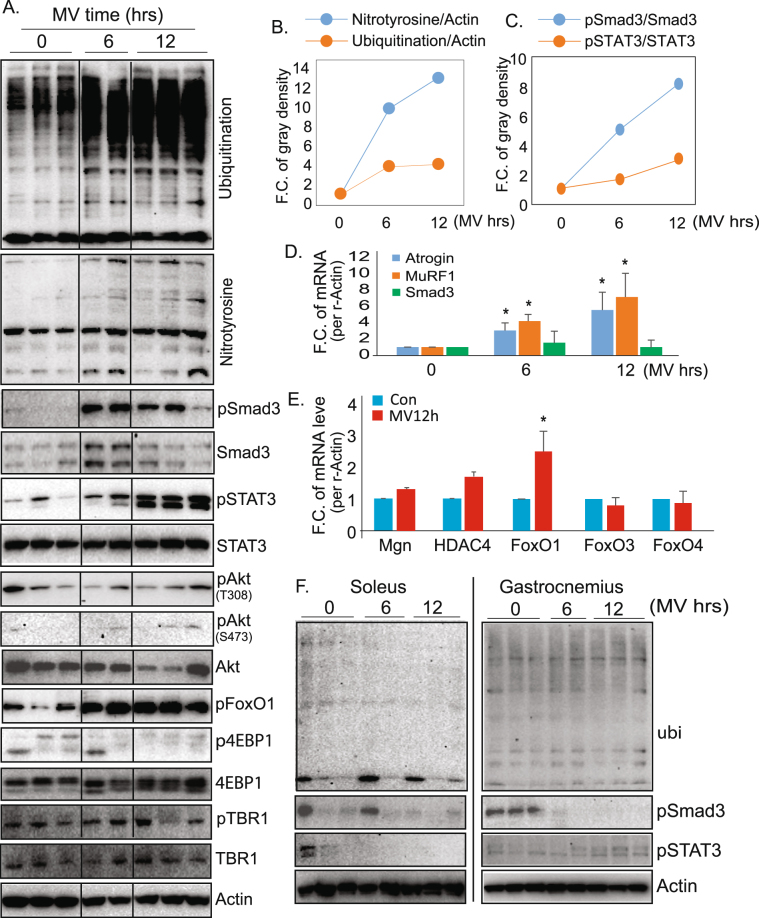
Mechanical ventilation rapidly activates Smad3 in diaphragm muscle and not limb muscle. (**A**) Western blot analysis of diaphragm tissue from rats ventilated for 6 and 12 h. Note the rapid protein oxidation and protein ubiquitination, accompanied by induction of phosphorylated Smad3, prior to the induction of STAT3 phosphorylation. (**B**) Quantitation of protein oxidation and protein ubiquitination in MV diaphragm over time. FC – fold change (n = 4 rats for each group). (**C**) Quantitation of Smad3 and STAT3 phosphorylation in MV diaphragm over time (n = 4 rats for each group). (**D**,**E**) mRNA expression by RT-PCR of genes implicated in VIDD in diaphragm after 6 and 12 h of MV (n = 3 rats for each group). (**F**) Western blot showing ubiquitination, Smad3 phosphorylation, and STAT 3 phosphorylation over time in limb muscles of MV rats. Note the absence of the time-dependent changes that occur in diaphragm muscle. Data information: In (**B**–**E**), data are presented as mean ± standard deviation. *p < 0.05 (Student’s t-test).

Given our interest in identifying activity-dependent sensors that couple diaphragm contraction with the known downstream pathogenic events that result in VIDD, we focused on the molecular changes that occur early after institution of MV (the 6 and 12-hour time points). We found that after as little as 6 h of MV, protein ubiquitination and protein oxidation are significantly induced in MV diaphragm (Fig. 2A and B).

We then examined the expression of previously identified activity-dependent regulators of protein turnover, such as Akt, FoxO and mTORC1 (Fig. 2A). Akt activity levels, revealed by the phosphorylation levels of Akt-T308 and S473, do not show significant changes during MV at these early time points. The phosphorylation levels of FoxO1 are, similarly, not suppressed by MV. At these early time points, FoxO1’s phosphorylation is, in fact, upregulated. Since phosphorylation of Akt and dephosphorylation of FoxO1 leads to the activation of Akt-FoxO proteolytic signaling, this regulatory pattern indicates that the posttranslational regulation of Akt-FoxO1 signaling is unlikely to be a major contributor in the initiation of protein ubiquitination and protein oxidation at these early time points. Similarly, we did not observe a significant change in the phosphorylation status of 4EBP1 (p4EBP1/4EBP1), indicating that mTORC1 also is not activated at this early stage. Meanwhile, the TGFb receptor type I (TBRI) is not regulated by MV at either the total protein level or its phosphorylation status (Fig. 2A). In contrast, the phosphorylation of STAT3, a previously identified regulator of VIDD, is dramatically upregulated at 12hr MV, but not at 6hr.

Most interestingly, Smad3, a transcription factor in the TGF-beta signaling pathway, is robustly (>8 fold) and rapidly (as early as 6hr) phosphorylated (Fig. 2A and C), similar to the time course of increased protein oxidation and protein ubiquitination (Fig. 2B) and also to the time course of expression of the E3 ubiquitin ligases, atrogin and MuRF1 (Fig. 2D). The mRNA levels of Smad3 remain unchanged during 12-hr MV, indicating that Smad3’s regulation is primarily at the posttranslational level rather than the transcriptional level at this early stage (Fig. 2D). We also measured the transcriptional expression of other potential activity-dependent regulators that have been previously identified in limb muscles (Fig. 2E). HDAC4, myogenin, FoxO3, and FoxO4 do not show significant changes at 12hr MV, but FoxO1 is moderately upregulated.

These molecular alterations associated with MV appear to be diaphragm-specific, and not systemic, as we did not observe activation of Smad3 (pSmad3) or STAT3 (pSTAT3) in hindlimb muscles (soleus and gastrocnemius), and the levels of protein ubiquitination also remain unchanged (Fig. 2F).

### Smad3 phosphorylation is regulated by muscle activity

**Figure 3:**
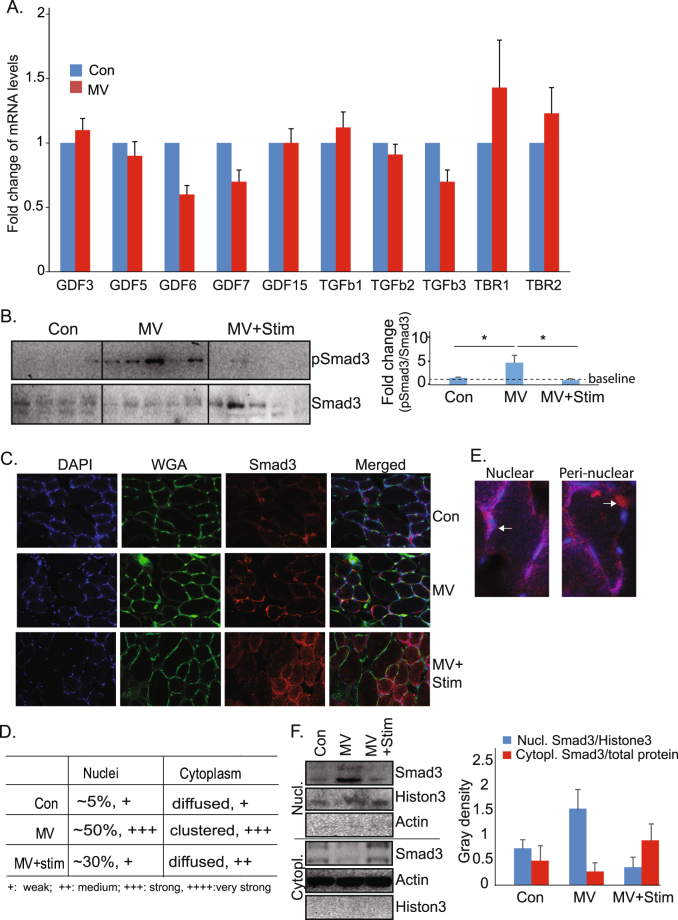
Smad3 is an activity-dependent sensor in diaphragm muscle. (**A**) Quantitation by RT-PCR shows no significant changes of members in TGFb super family, as well as the subunits in TGFb receptors, in 12 h MV diaphragm muscle (n = 4). (**B**) Western blotting (left panel) and quantitation (right panel) show that phosphorylation of Smad 3 is induced in diaphragm by MV and reversed by PNS (n = 3 rats per group). (**C**) Representative images of Smad3 immunostaining in diaphragm muscles of control, MV and MV + Stim rats. (**D**) Quantitation of the subcellular distribution of Smad3 (n = 3 rats, ~100 fibers/rat) in control, MV and MV + stim. (**E**) Representative images of the nuclear and perinuclear distribution of Smad3. (**F**) Nuclear enrichment of Smad3 in MV diaphragm is reduced by electrical stimulation. Western blot was performed and gray density measured to quantitatively (n = 3) show the subcellular localization of Smad3. Nuclear fraction of Smad3 was normalized to nuclear protein Histone 3, while cytoplasmic fraction of Smad3 was normalized to actin. Data information: In (**A**) and (**B**), data are presented as mean ± standard deviation. *p < 0.05 (Student’s t-test).

The noted increased phosphorylation of Smad3 could potentially be due to activation of the canonical TGFβ signaling pathway. It has, in fact, been reported that TGFβ receptor subunit I (TBRI) is upregulated in muscle after 3-days of denervation^45^. To test this, we measured the expression of TGFβ receptor subunits as well as of several other members in the TGFβ super-family. We did not observe these to be upregulated significantly (Fig. 3A). In contrast, we found that Smad3′s phosphorylation is regulated directly by electrical activity. MV-induced phosphorylation of Smad3 protein is nearly completely prevented by PNS (Fig. 3B).

In a further analysis of the subcellular localization of Smad3 in MV and MV + Stim diaphragm samples, we found that MV results in nuclear enrichment of Smad3, with about 50% of nuclei showing visible staining at an average intensity of “+++” by 12 hours. The control diaphragm samples show only approximately 5% of nuclei staining with Smad3 antibody at an average intensity of “+” (Fig. 3C and D). Electrical stimulation prevents the nuclear enrichment of Smad3, with MV+Stim samples demonstrating a more diffuse pattern of staining throughout the muscle fibers. It is worth noting that even in MV diaphragm, the clustered Smad3 is strongly stained not only in the nuclei themselves, but also in the perinuclear region in many fibers (Fig. 3E). This implies that perhaps nuclear enrichment is not the only way that phosphorylated Smad3 exerts its function. To confirm the nuclear enrichment of Smad3, we isolated the nuclear fraction from diaphragm muscles from control, MV, and MV/stimulation rats. This data (Fig. 3F) quantitatively shows that MV induces the nuclear enrichment of Smad3 in diaphragm muscles and that this is prevented by electrical stimulation.

### Smad3 activity regulates oxidative stress, the ubiquitin proteasome system, and the phosphorylation of STAT3 in cultured muscle cells

It is known that overexpression of Smad3 in limb muscle leads to muscle atrophy^46^. It is also known that Smad3 can regulate NOX4 and that it links to mitochondrial oxidative stress^47^. However, it has not been demonstrated that there is a link between Smad3 and the specific downstream effectors of VIDD in MV diaphragm. We thus examined if Smad3 directly participates in the regulation of these downstream effectors.

**Figure 4:**
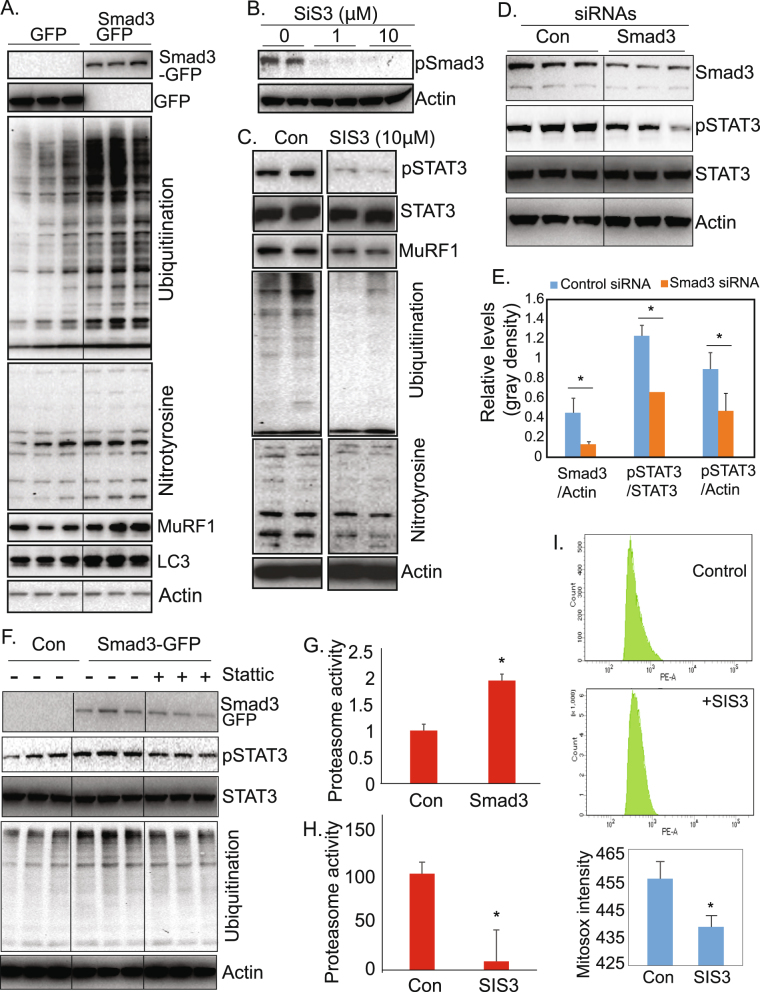
Smad3 is involved in the regulation of oxidative stress, proteasomal activity, and the phosphorylation of STAT3 in cultured muscle cells. (**A**) Western blots from cultured C2C12 cells transfected with control or Smad3 expression vectors show that protein oxidation, ubiquitination, and E3 ubiquitin ligase are upregulated in response to Smad3 overexpression. (**B**,**C**) Western blots from cultured C2C12 cells treated with Smad3 inhibitor SIS3 after differentiation. (**B**) A dose-dependent suppression of Smad3 phosphorylation by SIS3. (**C**) STAT3 phosphorylation and downstream proteins are significantly reduced in response to Smad3 inhibition (SIS3, 10 µM). (**D**,**E**). Silencing Smad3 with siRNA suppresses the phosphorylation of STAT3. D) Control and Smad3 SiRNAs (100 nM) were transfected into C2C12 cells. Three days after differentiation, cell lysates were subjected to Western blot analysis. (**E**) The gray density of the expressed proteins was quantitated by Image J (n = 3 samples per group). (**F**) Western blots from cultured C2C12 cells transfected with control or Smad3 expression vectors show that protein ubiquitination, and phosphorylation of STAT3 are upregulated in response to Smad3 overexpression, but were suppressed by the treatment of Stattic (10 µM), an STAT3 inhibitor. (**G**,**H**). Quantitation of protein oxidation and proteasomal activity after overexpression (**C**) or inhibition (**D**) of Smad3 in C2C12 cells (n = 6 samples per group). (**I**) MitoSOX intensity in C2C12 cell treated with Smad 3 inhibitor, SIS3, shows reduced generation of reactive oxygen species (n = 6 samples per group). Data information: In (**C**–**E**), data are presented as mean ± standard deviation. *p < 0.05 (Student’s t-test).

In cultured cells, we found that overexpression of Smad3 induces protein oxidation (revealed by the levels of nitrotyrosine) and protein ubiquitination, autophagy (revealed by LC3), as well as the expression of E3 ligases MuRF1, indicating that Smad3 activation is sufficient to induce oxidative stress and protein degradation (Fig. 4A). Further, treatment with the Smad3 inhibitor, SIS3, in cultured myotubes suppressed protein oxidation and protein ubiquitination, as well as the expression of E3 ubiquitin ligase (Fig. 4B and C). Interestingly, we found that STAT3 phosphorylation – which were shown to be an important step in the development of VIDD^29,30^ –is dependent on Smad3 activity. Inhibition of Smad3 activity with SIS3 leads to a dramatic reduction of STAT3 phosphorylation (Fig. 4C). Partial knockdown of Smad3 levels by Smad3 siRNA also leads to a reduced levels of pSTAT3 and nitrotyrosine, without affecting the STAT3 expression levels (Fig. 4D,E and Fig. S1A). There thus exists a previously unappreciated link between Smad3 and STAT3 activity. We further examined if STAT3 activity contributes to Smad3′s effect on protein degradation. Overexpressed Smad3 induces phosphorylated levels of STAT3, protein ubiquitination, and nitrotyrosine, but these induced levels were reduced by STAT3 inhibitor, Stattic (Figs 4F, S1B, S2). Therefore, STAT3 activation participates in Smad3-induced protein degradation.

In addition, we are able to demonstrate also that ubiquitin proteasomal activity is controlled by Smad3 (Fig. 4G and H). Overexpression or inhibition of Smad3 activity results in upregulation or suppression of proteasomal activity, respectively. Further, cultured myocytes treated with SIS3 for 24 hours at 10 µM, stained with MitoSOX, and subjected to flow cytometry show that inhibition of Smad3 activity with SIS3 reduces ROS generation (Fig. 4I).

### Smad3 activity regulates JAK-STAT and is required for MV-induced oxidative stress, ubiquitin proteasome activity, and reduced diaphragm contractility *in vivo*

The above *in vitro* data indicates that Smad3 activity regulates STAT3 phosphorylation and is able to regulate oxidative stress, protein oxidation and protein ubiquitination. However, *in vivo* studies were required to determine if Smad3 plays an important role in the early development of oxidative stress and protein degradation in the VIDD syndrome. We therefore tested the *in vivo* effect of Smad3 inhibition on ubiquitin proteasomal activity during short-term MV in rats.

**Figure 5:**
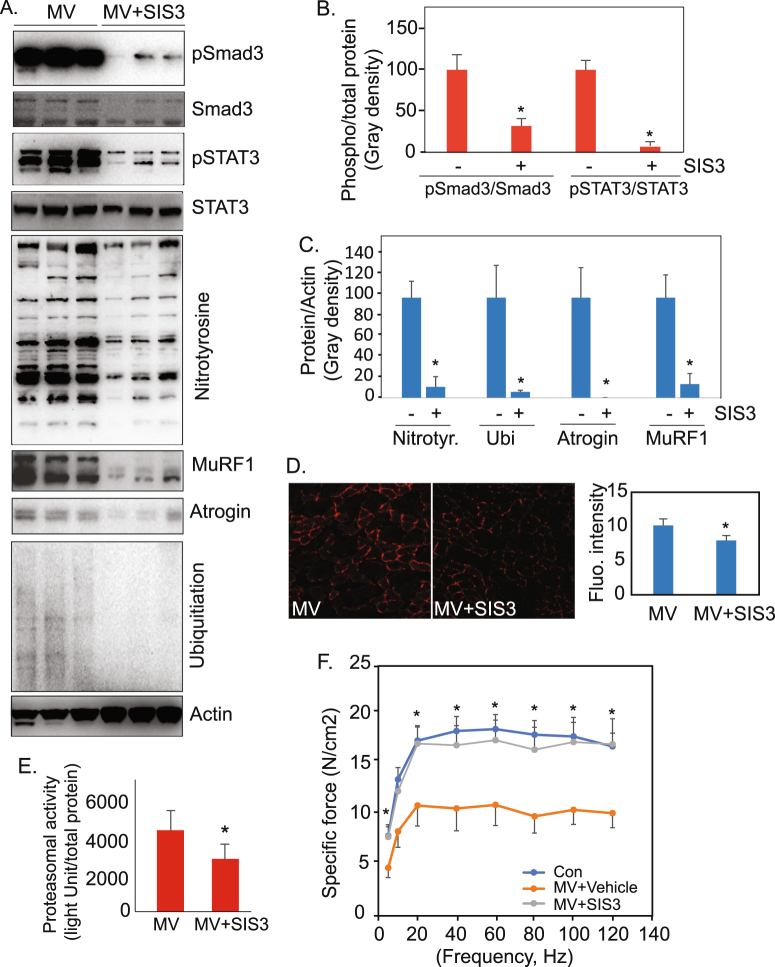
Inhibition of Smad3 suppresses oxidative stress, proteasomal activity, and contractile dysfunction in the diaphragm of rats subjected to mechanical ventilation. (**A**) Western blot shows that inhibition of Smad3 by intraperitoneal administration of SIS3 during MV in rats suppresses STAT3 phosphorylation, protein oxidation, ubiquitin ligase levels, and ubiquitination in MV diaphragm. (**B**) Quantitation of the phosphorylation levels of Smad3 and STAT3 in response to MV and MV with SIS3 treatment (n = 3 rats each group). (**C**) Quantitation of the levels of the ubiquitinated and oxidized proteins, and E3 ubiquitin ligases, in response to MV and MV with SIS3 treatment (n = 3 rats each group). (**D**) DHE staining of the diaphragm muscles from MV rats and MV rats with SIS3 treatment (n = 3 rats each group). (**E**) Quantitation of proteasomal activity (chymotrypsin-like) in MV diaphragm and MV diaphragm with intraperitoneal SIS3 treatment shows that *in vivo* Smad3 inhibition suppresses proteasomal activity (n = 3 rats each group). (**F**). Inhibition of Smad3 prevents the reduction in specific isometric contractile force that occurs in mechanically ventilated rats. Isometric force of diaphragm muscle strips was measured at Lo from control rats (n = 5) and from MV rats treated with either vehicle (n = 8) or the Smad3 inhibitor SIS3 (n = 6) at stimulation frequencies between 5 and 120 Hz. The specific force at each frequency was calculated and is shown (force-frequency curve). Values are means ± SE. Comparisons both of the entire curves, and of the means at each individual frequency except 10 Hz, indicate that Smad3 prevents the reduction in specific force caused by MV (*Indicates p < 0.05 for both control vs MV and for MV + SIS3 vs MV at any given frequency). At no frequency is there a significant difference between the control and MV + SIS3 groups. Data information: In (**B**–**E**), data are presented as mean ± standard deviation. *p < 0.05 (Student’s t-test). In (**F**), data are presented as mean ± standard error. *p < 0.05. Two-way ANOVA was used to determine differences between the entire force-frequency curves; one-way ANOVA followed by a post-hoc Tukey HSD Test was used to compare the differences between the means at each individual frequency.

Sprague-Dawley rats were subjected to MV for 12 hours, with or without intraperitoneal administration of the Smad3 inhibitor SIS3 (1 mg/kg/hr). Diaphragm muscle samples were harvested for protein analysis. SIS3 treatment dramatically suppressed MV-induced Smad3 activity as well as STAT3 phosphorylation in the muscle (Fig. 5A and B). Concomitantly, protein oxidation and ubiquitination, and the expression of E3 ubiquitin ligases, were all clearly reduced by the Smad3 inhibitor (Fig. 5C). Oxidative stress in MV diaphragm, revealed by DHE (dihydroethidium) staining, and proteosomal activity were also reduced with SIS3 administration (Fig. 5D and E).

We also measured diaphragm contractile properties to determine the effect of SIS3 treatment on diaphragm function. Isometric specific contractile force was measured in diaphragm muscle strips at Lo, at stimulation frequencies between 5 Hz and 120 Hz, in 3 cohorts of rats: control (non-ventilated), MV with vehicle, and MV with Smad3 inhibitor (SIS3). Inhibition of Smad3 with SIS3 effectively prevents the MV-induced reduction in contractile force at nearly every frequency of stimulation across the force-frequency curve (Fig. 5F), indicating a critical role for Smad3 in the evolution of VIDD. We also searched for the development of diaphragm fiber atrophy between control and MV rats. We did not observe a significant difference in fiber CSA between the groups at the 12 hour duration of our experiments (data not shown).

## Discussion

It is well-established that prolonged MV leads to diaphragm muscle atrophy and reduced specific force of contraction, and this is very likely to be a major factor underlying prolonged ventilator-dependence in many intensive care unit patients. MV-induced diaphragm weakness has been described in detail as a progressive, time-dependent problem that occurs through the activation of series pathogenic events which include the induction of oxidative stress and the activation of the proteasome system^15–17^. Activated JAK-STAT signaling in MV has been noted to be a critical part of the signaling cascade that leads to this oxidative stress and protein degradation^29,30^.

It has been a reasonable hypothesis that the elimination (or at least major reduction) in diaphragm electrical activity and/or mechanical displacement that occurs during full ventilator support in MV serves as the upstream initiator of the signaling cascade that ultimately results in diaphragm atrophy/dysfunction^9,25^. However, the link between the physical changes associated with diaphragm disuse during MV and the subsequent molecular alterations has not been defined. In the current study, we chose to interrogate the early time points after institution of MV in order to try to identify molecular signals that might serve as this physico-molecular couple.

We identify Smad3 as an activity-dependent sensor which responds rapidly to MV-induced muscle inactivity by altering its phosphorylation status. We further demonstrate that Smad3 activity regulates protein degradation and oxidative stress both *in vitro*, in cultured muscle cells, and *in vivo*, in a model of rat MV. We used both DHE and nitrotyrosine to detect the levels of oxidative stress. Increased levels of nitrotyrosine indirectly reflect levels of the oxidant peroxynitrite. While the formation of peroxynitrite from nitric oxide and superoxide radicals apparently reduces intracellular superoxides and thus could be considered an antioxidant process, the proteins subsequently targeted by the peroxynitrote are likely functionally altered, and they will ultimately degraded by 20 S proteasome.

More importantly, we show that Smad3 activity is required for the activation of STAT3 during MV, thereby linking the current findings to STAT3 signaling, which we and others^29,30^ have previously identified to be a critical regulator of VIDD (Fig. 5F). Lastly, we show that Smad3 inhibition in a rat MV model effectively prevents VIDD. The Smad3 inhibitor, SIS3, is known to selectively inhibit Smad3 phosphorylation without affecting other signaling pathways such as MAPK/p38, ERK or PI3K^48^. We further validated the effect of Smad3 inhibition using siRNA. The results were similar to those with SIS3. As a whole, these findings further our understanding of the pathogenesis of VIDD, and they suggest the possible development of a clinical intervention based upon inhibition of Smad. Such a drug, if delivered to mechanically ventilated patients upon ICU admission, may be able to reduce the rate of prolonged ventilator dependence and subsequent cascading complications and deaths.

During full ventilator support, diaphragm muscle is rendered inactive^35–38^; the phrenic nerve is not firing, but it remains intact. The molecular mechanisms coupling muscle inactivity and subsequent molecular and phenotypic changes have been actively studied, but only in limb muscle denervation models – where the nerve of interest is physically divided. Denervation-associated inactivity activates several signaling pathways that can regulate muscle gene expression, including the HDAC4-Dach2-Myogenin^49–51^ and mTORC1-FoxO pathways^52^. Interestingly, we find that gene expression levels in these pathways are unaltered at the early stages after institution of MV, indicating that these molecules likely do not play initiating roles in VIDD. Since diaphragm disuse during MV, where there is an intact phrenic nerve, is not directly analogous to denervation, it is perhaps not surprising that in this model we identify, early after MV, a role for a novel activity-dependent sensor, Smad3.

Smad3 is a transcription factor whose transactivity is known to be regulated by the TGFβ signaling pathway through the interaction between TGFβ receptor and ligands such as TGFs and GDFs. These interactions lead to the activation of TGFβ receptor kinase (serine threonine kinase) and thereby the activation (phosphorylation) of Smad3. Interestingly, expression of TGFβ receptor subunit I was demonstrated to be activity-dependent in cultured myotubes, and this links to the activation of Smad2/4, but Smad3 was not examined^45^. However, in MV diaphragm, the ligands and receptor subunits in the TGFβ signaling pathway did not show early changes at either the mRNA level (for the TGFβ receptor subunits and ligands) or the protein level / phosphorylation status (for the type I TGFβ receptor [TBRI]). It is thus likely that Smad3 in this scenario is regulated by mechanisms other than TGFβ receptor-ligand interaction.

Perhaps Smad3 during MV is phosphorylated by non-canonical TGF-b signaling pathways such as p38 MAP kinase, angiotensin II type I receptor (AT1), or others^53^. Interestingly, in a previous study, inhibition of AT1 achieved promising results in preventing VIDD^54^. This effect may link to Smad signaling, since Smads are indeed non-canonical downstream targets of AT1^55,56^. Mitochondrial dynamic disturbance that has been observed at 6 hrs of MV in a mouse model^57^ is an early event as well. Since Smad3 is related to the induction of oxidative stress, perhaps, Smad3′s activation also contributes to this early dynamic and morphological change of mitochondria. In addition, calcium may be a regulator of Smad3, since muscle activity regulates calcium release and the activation of calcium-related kinases such as CaM Kinases. Intracellular calcium release from sarcoplasmic reticulum (SR) is regulated by the ryanodine receptor, and this receptor has also been reported to be dysfunctional in VIDD^58^. Blockage of this SR receptor, however, had mixed results^58,59^. The involvement of calcium related kinases and the ryanodine receptor in Smad3 activation remains to be further explored.

JAK-STAT signaling is closely linked to diaphragm muscle dysfunction in MV. Increased phosphorylation of STAT3 has been observed in rat and human MV diaphragm muscle, and inhibition of JAK-STAT signaling substantially prevents the oxidative stress and protein degradation associated with MV as well as the reduction in diaphragm contractility^29,30^. In the current study, we further the understanding of JAK-STAT in VIDD by identifying link between Smad3 and STAT3. This link bridges the TGFβ and interleukin (IL) families. Smad3 appears to be upstream of STAT3, as Smad3 activation occurs earlier after institution of MV than does STAT3 phosphorylation, and inhibition of Smad3 leads to inhibition of STAT3.

It is possible that Smad3 and STAT3 may govern common downstream targets–for example NOX4-mediated mitochondrial dysfunction/oxidative stress^60,61^ and ubiquitin proteasome-dependent protein degradation^30^. Consistently, it has been observed that overexpression of either Smad3 or STAT3 is able to induce muscle fiber atrophy in skeletal muscle^46,62^, and excess Smad signaling has been linked to skeletal muscle dysfunction^63^. Smad2/3 is required for denervation- and immobilization-induced muscle atrophy^64^. The phosphorylation levels of Smad3 are also significantly elevated in denervated gastrocnemius muscle (at 1 week), with a concomitant induction of total Smad3 protein^64^. This indicates that though Smad3 is commonly activated in these different models of muscle inactivity, the regulatory pattern (e.g., time of activation, ratio of the phosphorylated over total protein) varies from model to model.

The findings we report here advance the understanding of the basic regulatory mechanism that occur early in inactive, mechanically ventilated diaphragm muscle. The fact that Smad3 is an important and crucial early responsive, activity-dependent sensor renders this a potential drug target to prevent VIDD and thus reduce rates of prolonged ventilator dependence.

## Materials and Methods

### Mechanical ventilation and SIS3 *in vivo* drug treatment

Animal experimental protocols were approved by the Institutional Animal Care and Use Committee in VA Palo Alto Healthcare center, and experiments were carried out in accordance with relevant guidelines and regulations. Mechanical ventilation was performed on tracheotomized Sprague-Dawley rats (male, ~400 to 500 g; age 4–6 month). Rats were anesthetized by intraperitoneal injection of pentobarbital (65 mg/kg). When they reached the anesthetic plane, we shaved and prepared the neck sterilely and made an L-shaped incision to open the skin from the midline to the right side, allowing exposure of the right jugular vein and trachea. The jugular vein was cannulated with a 22 gauge IV catheter, which was used for delivery of fluid (normal saline) and pentobarbital (5 mg/ml) to maintain homeostasis and absence of diaphragm contractions. Tracheotomy was then made and an intubating catheter (18 gauge) inserted. This was connected to small animal ventilator (SAR830, Harvard Apparatus). Rats were ventilated at 80 breaths/min, with a tidal volume of 0.6 ml/100 g body weight. PEEP was set at 1 cm H2O. We documented in a previous series of 8 animals that these settings consistently allowed long-term ventilation without development of hypoxia, acidosis (lower than 7.35), alkalosis (higher than 7.45), or hemodynamic instability. Control rats of MV were fasted during the MV period.

While ventilated, the animals′ vital signs were recorded every 15 min. Heart rate and oxygen saturation were monitored with a small animal pulse oximeter (Starr Life Sciences). Heart rate was maintained at 325 + /-25/min. Pentobarbital was delivered intravenously when the heart rate rose above 350/min, as we had learned in our previous experiments that this rise often portended subsequent spontaneous inspiratory efforts. Temperature was monitored with an anal temperature probe (Harvard Apparatus) and maintained at 36.5+/−0.5 degree by intermittent use of a heating blanket. Total fluid was maintained at about 3.5 ml to 4 ml/hr. Every 15 min, the animals were gently rotated side-to-side, their hindlimbs passively stretched, and the urinary bladder gently pressed in order to evacuate it. Animals were ventilated for 12 hours, at which time the animals were sacrificed and diaphragm and other muscles harvested for analysis. If more than one episode of spontaneous diaphragmatic contractions occurred in MV rats, or if a single episode lasted for more than 20 seconds before suppression by pentobarbital administration, then that animal was not further studied or included in the cohort.

In MV rats treated with the Smad3 inhibitor SIS3. SIS3 was injected intraperitoneally at 1 mg/kg/hr beginning one hour before the initiation of MV. Control rats of SIS3-treated MV rats were MV rats given only intraperitoneal injection of vehicle.

### Phrenic nerve stimulation (PNS)

In the cohort of animals receiving PNS, after inserting the jugular catheter and the tracheotomy, the right phrenic nerve was identified in the neck, carefully dissected circumferentially, and hooked in tension-free fashion with a fine platinum electrode. The grounding electrode was inserted into the ipsilateral pectoralis muscle. Phrenic nerve stimulation was performed periodically during MV (Fig. 1A and B and description in Results section), with each PNS-induced inspiration replacing one MV-dependent inspiration, once every 4 breaths (coinciding with a single-breath pause in the ventilator-delivered breaths). Electrical stimulation was controlled by Power Lab (ADInstruments). The stimulation voltage was set at 0.5 to 1 V, beginning at the lowest setting, and increasing to the point at which a maximal diaphragm contraction was visible by abdominal displacement. Diaphragm muscle was stimulated with a train of square waves with a stepwise increased intensity for a total 0.375 seconds. This MV and stimulation pattern was maintained for 12 hours, at which time the animals were sacrificed and the stimulated hemi-diaphragm muscle (the diaphragm ipsilateral to the stimulation) was harvested for analysis.

### Measurement of muscle isometric force

Diaphragm contractile function was determined using diaphragm strips maintained *ex vivo*, as described previously^29,30^. Briefly, upon completion of the study, the entire diaphragm with attached ribs was removed and transferred to a dissecting dish containing Krebs-Hensleit physiological solution aerated with 95% O_2_ / 5% CO_2_ gas. A muscle strip was dissected from the midcostal region of the diaphragm, including a portion of the central tendon and ribcage. The strip was mounted vertically, with one end fixed to an isometric force transducer on a tissue organ bath system (Radnotti), and immersed in the same solution. After a 15-min equilibration, diaphragm strips were stimulated with a Biphasic Stimulator controlled by Powerlab (ADInstruments). Isometric specific contractile force was measured at the optimal muscle length (Lo), the length at which maximal force is obtained. Lo was determined by stimulating the muscle at a supramaximal voltage (10-V, square wave), and systematically adjusting the muscle length. The force was then measured at varied stimulation frequencies: 5, 10, 20 40, 60, 80, 100, 120HZ with 120 repeats. Specific force was then calculated by dividing muscle force by the muscle cross-sectional area (CSA). Total muscle CSA at right angles to the long axis was determined by the following calculation: total muscle CSA (mm^2^) = [muscle mass/(fiber length at Lo × 1.056)], where 1.056 is the density of muscle (in g/cm^3^). Two-way analysis of variance (ANOVA) was used to test for significant differences between the 3 different groups over the entire force-frequency curve. One-way ANOVA with post-hoc Tukey Honestly Significant Difference (HSD) Test was used to determine the significant differences between the means between Control, MV, and MV + SIS3 (our Smad3 inhibitor) at each specific frequency. p < 0.05 was considered significant.

### Muscle Cross-Sectional Area Measurement

Freshly frozen muscle samples were sectioned at 14-μm thickness, stained with WGA–Alexa Fluor 488 (Invitrogen) as per the manufacturer’s instructions, mounted in Prolong Gold anti-fading reagent, and imaged by confocal fluorescent microscopy. CSA was measured using Fiji (http://fiji.sc/wiki/index.php/Fiji) software, an enhanced version of Image J. Three different regions from each section (total ∽300 fibers/sample) and 3 consecutive sections (repeats) were processed. The detailed measurement procedure is available upon request. One way ANOVA was used to evaluate the statistical significance between control (n = 8), MV (n = 8) and MV+SIS3 (n = 6). *P* < 0.05 was considered significant.

### Protein and RNA expression

Protein expression levels were determined by Western blot analysis. Protein was extracted from muscle tissues or cultured cells with RIPA buffer (10 mM Tris-Cl (pH 8.0), 1 mM EDTA, 1% Triton X-100, 0.1% sodium deoxycholate, 0.1% SDS, 140 mM NaCl), supplemented with phosphatase inhibitors (Phosphatase cocktails 1 and 2, Sigma) and protease inhibitors (Complete Mini, Roche). Protein concentrations were determined by Dc Protein Assay (BioRad) following the manufacturer’s protocol. 15 to 30ug of protein was separated by SDS-PAGE in 4–12% Bis-Tris and transferred to nitrocellulose membranes (NuPage system, Invitrogen) for Western blot analysis. Membranes were incubated with antibodies (1:500 or 1:1000 in 5% BSA in TBST). Membranes were then treated with SuperSignal West Pico Chemiluminescent Substrate (Thermo Fisher Scientific) for 5 minutes, and the resulting images were captured by the ChemiDoc XRS System (BioRad, Hercules, CA) for analysis. Between each step, the membranes were washed 3 times for 5 to 10 minutes with 0.1% TBS Tween. Primary and secondary antibodies were purchased from Cell Signaling Technologies.

mRNA expression levels were examined by quantitative PCR. RNA samples were extracted by Trizol (Invitrogen) after shearing the samples with Omni TH tissue homogenizer (Omni International). RNA concentration and purity were measured with a spectrophotometer (NanoDrop, Invitrogen). Reverse transcription of the RNA samples was performed using SuperScript II Reverse Transcriptase kit (Invitrogen) according to manufacturer’s instructions. mRNA expression levels were measured with real time PCR using Absolute blue SYBR Green ROX Mix (Thermo Fisher Scientific) and 7900HT Fast Real-Time PCR system (Applied Biosystems). Gamma-actin was used as a control to calculate the relative Ct values. All reactions were performed in triplicate.

### Cell culture, transfection, flow cytometry, and Western blot analysis

C2C12 myoblasts were cultured and for Smad3 inhibitor experiments treated with SIS3 at 10µM for 24 hours. Cells were then digested for flow cytometry. Flow cytometric studies were performed by standard methods with an LSR II flow cytometer unit (Benton Dickinson). For our experiment, cells were stained with mitoSOX (Thermo Fisher Scientific). After staining, the cells were filtered through a cell strainer and 10,000–1,000,000 events were acquired for each sample. Flow cytometry data were analyzed with FACSDiva software (Benton Dickinson). For the Smad3 overexpression experiment, the Smad3 expression vector (pMX-IRES-GFP-Smad3, a gift from Dr. Xuedong Liu, University of Colorado) was transfected for 3 days and cell lysate were then collected for Western blot analysis. Western blot analysis was performed with standard protocols, with primary antibodies purchased from Cell Signaling Technology. Full-length blots are included in the Supplementary Information.

Negative control siRNAs and Smad3 siRNA were purchased from Thermo Fisher Scientific. 100 nM siRNAs was transfected into C2C12 cells (~90% confluency) by Lipofectamine 3000 (Thermo Fisher Scientific). One day later, C2C12 cells were induced to differentiation in DMEM medium with 5% horse serum. Samples were then harvested for analysis 3 days after differentiation.

Primary antibodies against atrogin/MAFbx-1 and MuRF1 were purchased from Santa Cruz Biotechnology (Santa Cruz, California). Smad3 antibodies were purchased from both Cell Signaling Technologies (Danvers, Massachusetts) and Cayman Chemicals (Ann Arbor, Michigan). The rest of the primary antibodies and secondary antibody were purchased from Cell Signaling Technologies (Danvers, Massachusetts).

### Dihydroethidium (DHE) staining

DHE (Dihydroethidium) staining was performed as described previously^23^. Briefly, snap-frozen human diaphragm samples were sectioned on a cryostat at 20 μm. Freshly prepared 10 μM DHE/PBS solution (Invitrogen) was added to the cryosections and incubated for 10 min in a dark chamber. The reaction was stopped by washing 3 times in 1 x PBS. Slides were then fixed with 4% paraformaldehyde, mounted in ProLong Gold anti-fading reagent (Invitrogen), and imaged by a Leica confocal microscopy.

### Isolation of cytoplasmic and nuclear fractions

Fresh diaphragm muscle tissue was rinsed with cold PBS and then homogenized in homogenization buffer (250 mM sucrose, 50 mM Tris-HCL pH7.4, 5 mM MgCl2, supplemented with protease and phosphatase inhibitors cocktail) with a homogenizer (Omni International, TH-01). After incubation on ice for 30 min, the homogenates were centrifuged at 800 g for 15 min to separate the crude supernatant and pellet. This crude supernatant was further centrifuged at 14,000 g for 10 min to further remove the insoluble cell debris. The final supernatant from this centrifugation was saved as the cytoplasmic fraction. The crude pellet was re-suspended with the above homogenization buffer and centrifuged at 800 g for 15 min. After 3 repeats of this cycle of resuspension and centrifugation, the washed pellets were lysed with RIPA buffer. After 14, 000 g centrifugation, the supernatant of the lysate was saved as nuclear fraction.

### Proteasome activity assay

Total protein was extracted from diaphragm muscle tissue with 1X PBS buffer and subjected to proteasomal activity assay according to the manufacturer’s instructions (Promega).

## Electronic supplementary material

Supplementary Information
