# Supplementary material for: Smad3 initiates oxidative stress and proteolysis that underlies diaphragm dysfunction during mechanical ventilation
Source: Sci Rep. 2017 Nov 6;7:14530. doi: 10.1038/s41598-017-11978-4 (PMC5673963; doi:10.1038/s41598-017-11978-4)

Supplementary information

Smad3 initiates oxidative stress and proteolysis that underlies diaphragm dysfunction during mechanical ventilation

Huibin Tang[1,2], Catherine Kennedy[1,2,§], Myung Lee[1,2], Yang Gao[1,2], Hui Xia[1,2,§], Francesca Olguin[1,2], Danielle A. Fraga[1,2], Kelsey Ayers[1], Sehoon Choi[1,2,§], Michael Kim[1,2], Amir Tehrani[3], Yasser A. Sowb[3], Thomas A. Rando[2,4], and Joseph B. Shrager[1,2*]

[1]Division of Thoracic Surgery, Department of Cardiothoracic Surgery, Stanford University School of Medicine, CA, USA; [2]VA Palo Alto Healthcare System, CA, USA; [3]Respiratory Management Technologies, LLC., CA, USA; [4]Paul F. Glenn Laboratories for the Biology of Aging and Department of Neurology and Neurological Sciences, Stanford University School of Medicine, CA, USA

Supplementary figure legend:

Fig. S1. Quantitative result of the effect of Smad3 on STAT3 signaling. Gray density was measured by image J. The relative levels of phosphorylated protein were calculated by normalizing to actin or total protein. One way ANOVA was used to compare the difference,*$p<0.05$.

Fig. S2. The Smad3-dependent regulation of the intracellular levels of nitrotyrosine. A) Western blots from cultured C2C12 cells transfected with control or Smad3 expression vectors show that nitrotyrosine are upregulated in response to Smad3 overexpression, but were suppressed by the treatment of Stattic (10μM), an STAT3 inhibitor. Quantitative changes were shown at the left panel by gray density of the expressed proteins (quantitated by Image J (n=3 samples per group)). Actin (shown in Fig. 4F) was used for normalization of equal loading. One way ANOVA was used to examine the difference of the mean. *$p<0.05$. B) Silencing Smad3 with siRNA suppresses the levels of nitrotyrosine. Control and Smad3 SiRNAs (100nM) were transfected into C2C12 cells. Three days after differentiation, cell lysates were subjected to Western blot analysis. Actin (shown in Fig. 4D) was used for normalization of equal loading. Quantitative changes were shown at the left panel by the gray density of the expressed proteins (quantitated by Image J (n=3 samples per group)). Student t test, *$p<0.05$.

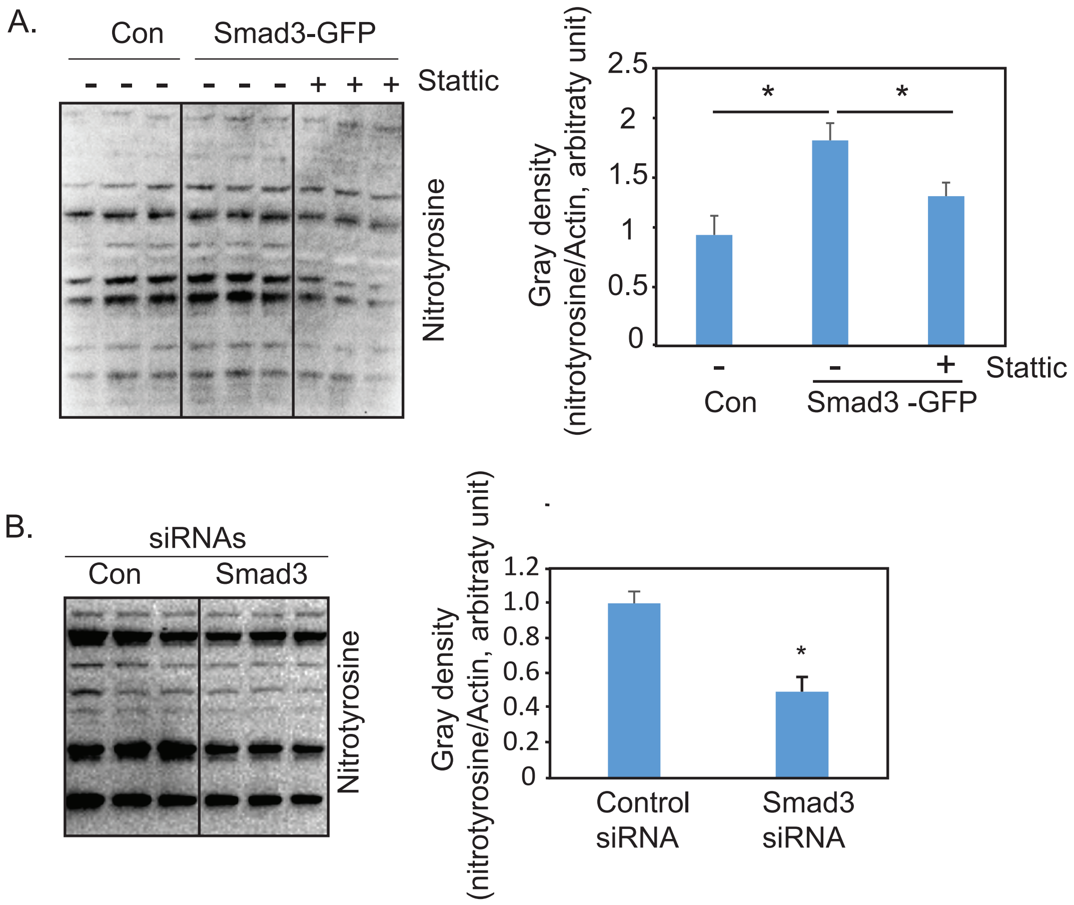
A.
Con
Smad3-GFP
- - - - - - + + +
Stattic
Nitrotyrosine
Gray density
(nitrotyrosine/Actin, arbitraty unit)
2.5
2
1.5
1
0.5
0
*
*
- - +
Stattic
Con
Smad3 -GFP
B.
siRNAs
Con
Smad3
Nitrotyrosine
Gray density
(nitrotyrosine/Actin, arbitraty unit)
1.2
1.0
0.8
0.6
0.4
0.2
0
*
Control
siRNA
Smad3
siRNA

Fig. S1

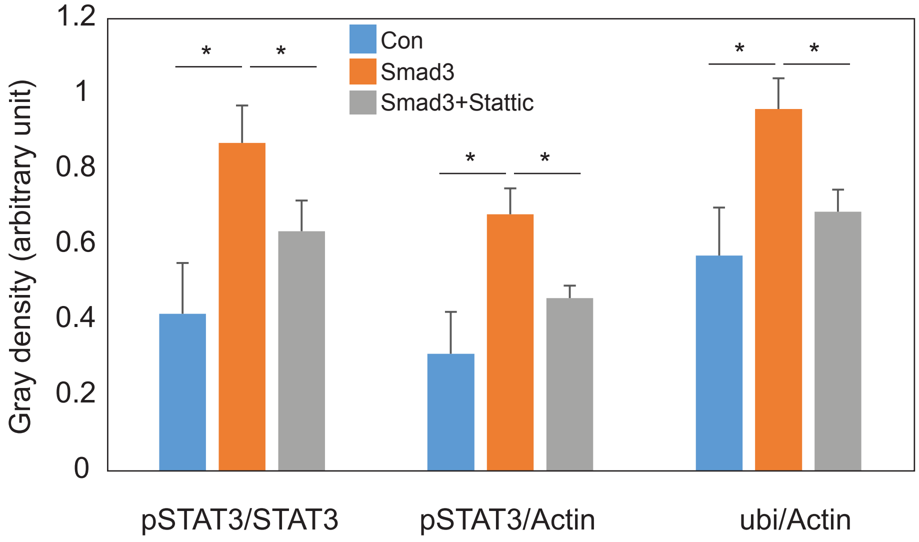

1.2
1
0.8
0.6
0.4
0.2
0
Gray density (arbitrary unit)
Con
Smad3
Smad3+Stattic
*
*
*
*
*
*
pSTAT3/STAT3
pSTAT3/Actin
ubi/Actin

Fig. S2

Supplementary info for the western blots used in Fig. 1C

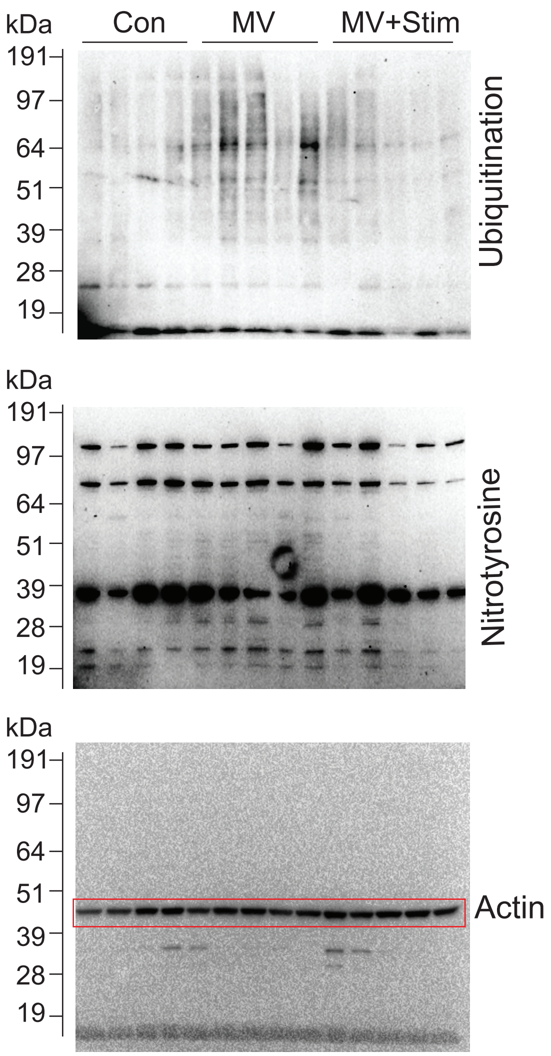

Supplementary info for the western blots used in Fig. 2A

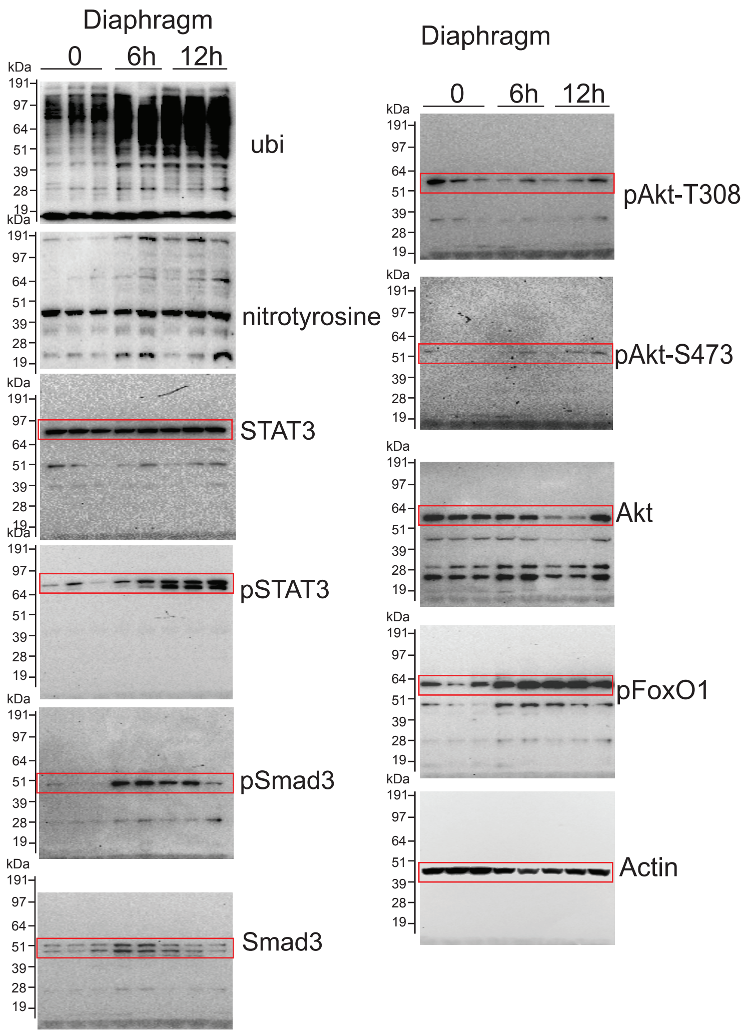

Fig. 2F

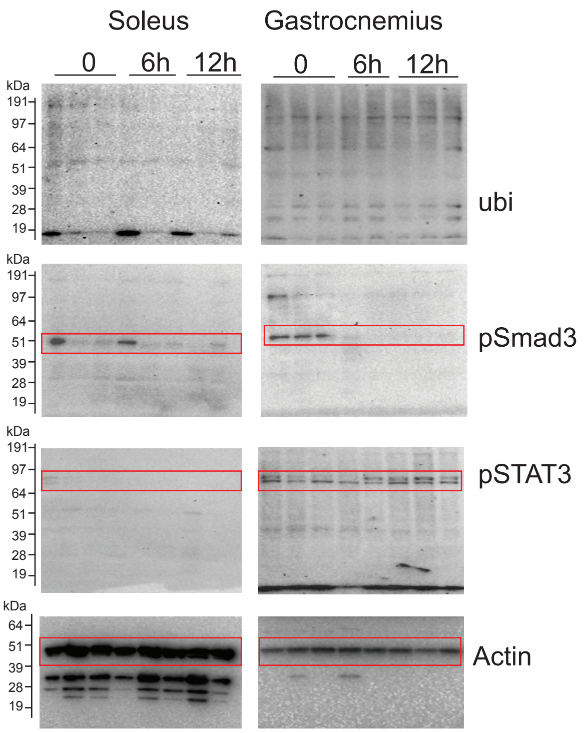

Supplementary info for the western blots used in Fig. 3B

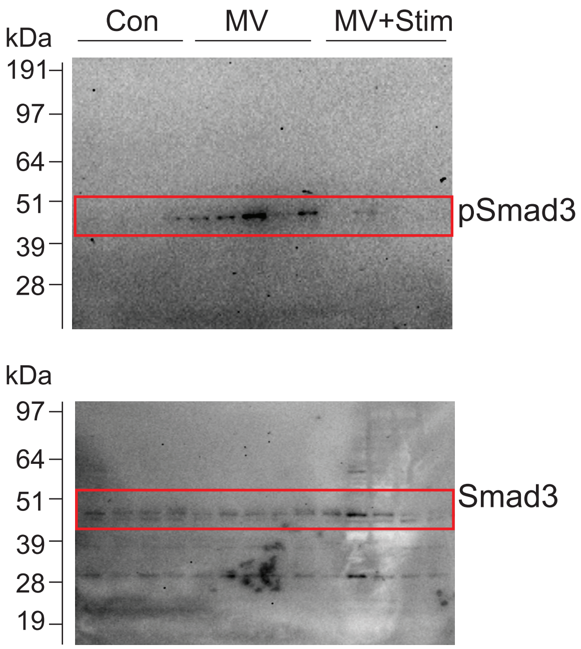

Supplementary info for the western blots used in Fig. 4

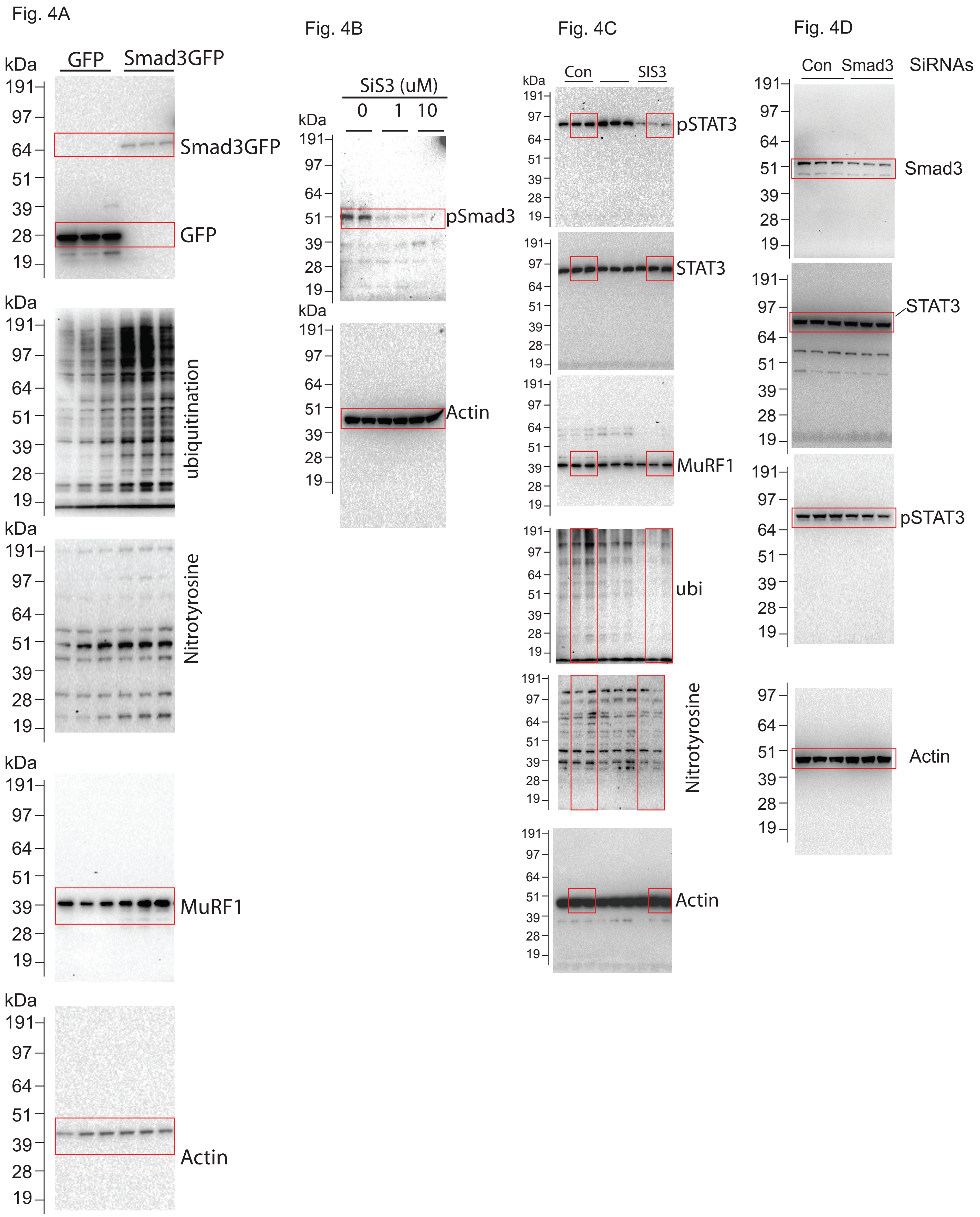

Supplementary info for the western blots used in Fig. 4

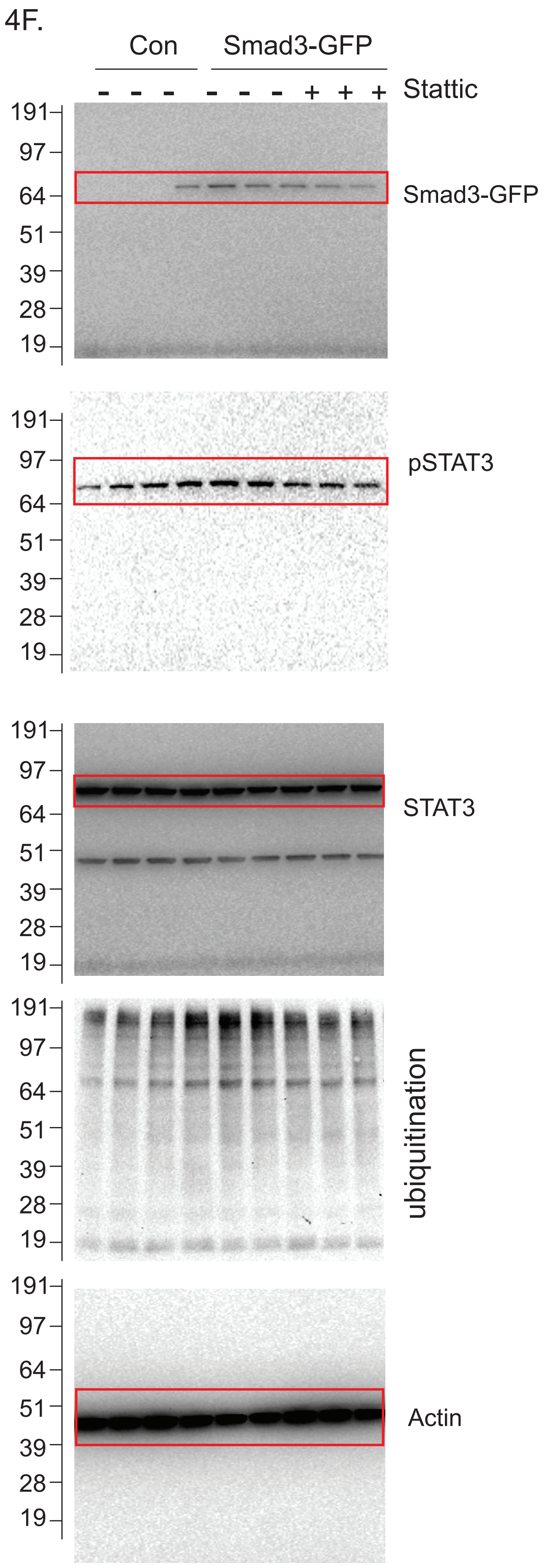

Supplementary info for the western blots used in Fig. 5A

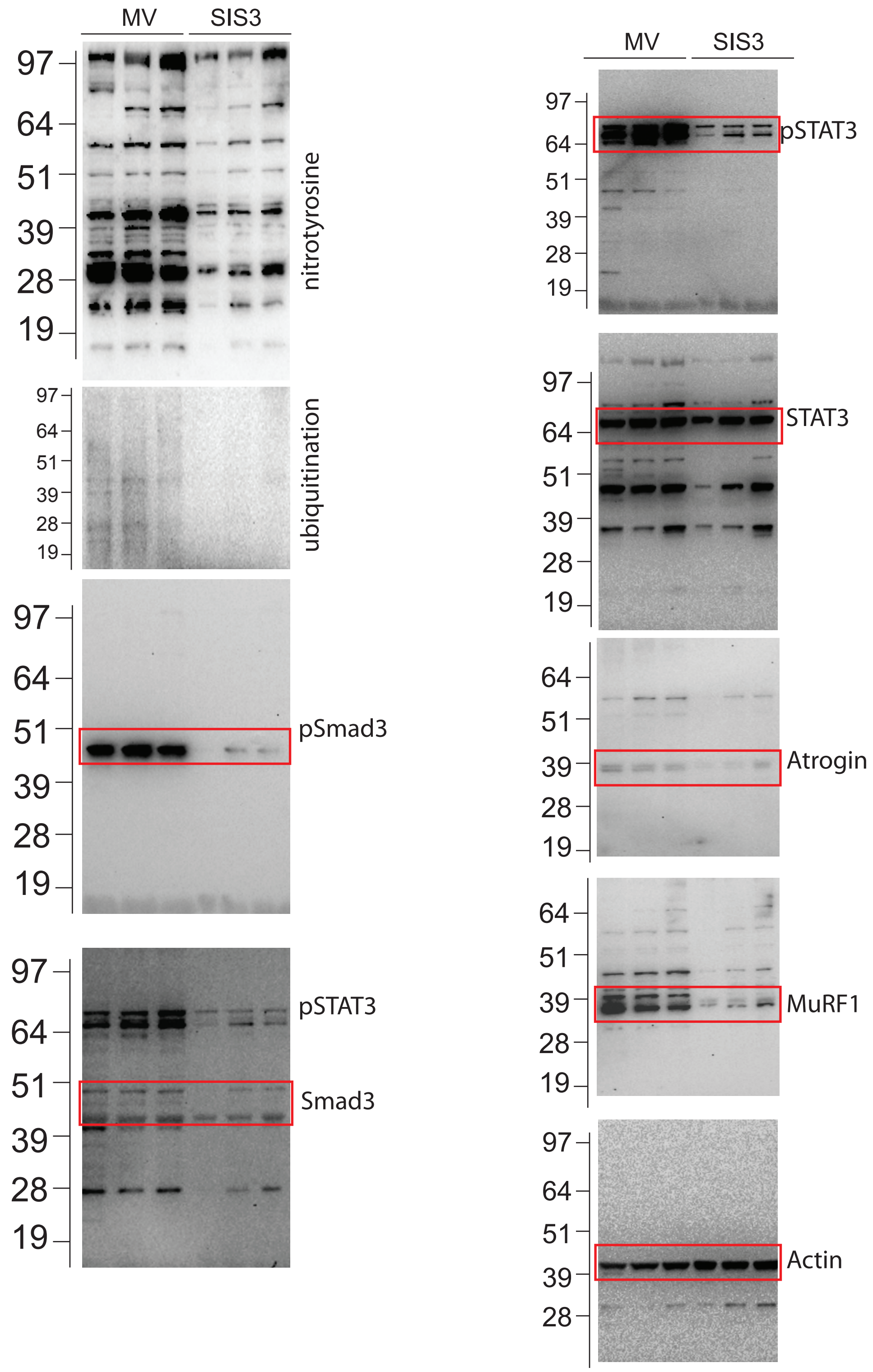

Supplement: Supplementary file 1 — Supplementary Information [file 41598_2017_11978_MOESM1_ESM.pdf]
